# Molecular and morphological evidence for the identity of two nominal species of *Astegopteryx* (Hemiptera, Aphididae, Hormaphidinae)

**DOI:** 10.3897/zookeys.833.30592

**Published:** 2019-03-25

**Authors:** Qiang Li, Jiamin Yao, Lingda Zeng, Xiaolan Lin, Xiaolei Huang

**Affiliations:** 1 State Key Laboratory of Ecological Pest Control for Fujian and Taiwan Crops, College of Plant Protection, Fujian Agriculture and Forestry University, Fuzhou 350002, China Fujian Agriculture and Forestry University Fuzhou China

**Keywords:** DNA barcoding, Hormaphidinae, integrative taxonomy, species delimitation

## Abstract

The morphology of many insect species is usually influenced by environmental factors and therefore high phenotypic variation exists even within a species. This causes difficulty and uncertainty in species taxonomy, which can be remedied by using molecular data and integrative taxonomy. *Astegopteryxbambusae* and *A.bambucifoliae* are currently regarded as two closely related aphid species with similar bamboo hosts and overlapping distributions in the oriental region. However, in practice it is hard to distinguish between them. By incorporating molecular data from four mitochondrial and nuclear genes as well as morphological information from an extensive collection of live specimens, the present study indicates that *A.bambucifoliae* is a junior synonym of *A.bambusae*. The data also indicate that large-scale geographic patterns of population differentiation may exist within this species.

## Introduction

For many insect groups, morphology is influenced by environmental factors. For example, aphids are a plant-feeding group with extremely high phenotypic plasticity across space and time, which can be influenced by different factors such as host plant (Wool and Hales 1997; Margaritopoulos et al. 2000), associated ant species (Yao 2012), climate and temperature (Blackman and Eastop 1994), as well as geography (Madjdzadeh and Mehrparvar 2009). In traditional insect taxonomy, species identification depends heavily on specimen morphology, and many species are first described based on only a small number of samples (Winston 1999; Eastop and Blackman 2005). However, for species with high intraspecific morphological variation, small samples from restricted areas and times cannot represent the complete range of morphological variation. This can cause difficulty and uncertainty in species delimitation, so that synonymies inevitably occur in taxonomy (Eastop and Blackman 2005; Meier 2017). Fortunately, new types of data yielded by new technologies such as DNA barcoding (Hebert et al. 2003; Foottit et al. 2008) and integrative taxonomic practices (Schlick-Steiner et al. 2010) can help to solve these problems and improve the quality and efficiency of taxonomy (Turčinavičienė and Rakauskas 2009; Jensen et al. 2010; Heethoff et al. 2011).

The genus *Astegopteryx* is an oriental aphid group with more than twenty species, and is the largest genus in the tribe Cerataphidini (Hemiptera, Aphididae, Hormaphidinae) (Blackman and Eastop 2018; Favret 2018). Some species of *Astegopteryx* have host alternation between their primary host plants, *Styrax* (Styracaceae) trees, on which they form multiple-cavity galls, and secondary host plants, mainly bamboos and palms (Kurosu and Aoki 1991; Aoki and Kurosu 2010; Huang et al. 2012; Blackman and Eastop 2018). However, many species can live exclusively on their secondary host plants with parthenogenetic reproduction (Blackman and Eastop 2018) and display variable morphology (Noordam 1991; Stern et al. 1997). In the taxonomic history of this genus, due to morphological variation between generations on different host plants (e.g. primary and secondary hosts) and even within generations (Aoki and Kurosu 2010), as well as species description on the basis of limited sampling, many synonyms have been created (Blackman and Eastop 1994; Favret 2018). Two currently valid species, *A.bambusae* (Buckton, 1893) and *A.bambucifoliae* (Takahashi, 1921), occur simultaneously on similar bamboo hosts and have overlapping distributions in the oriental region (Noordam 1991; Blackman and Eastop 1994; Qiao et al. 2018). These species have been distinguished mainly by differences in color and appearance in life, as well as some differences in morphology of antennae and wax glands in mounted specimens (Blackman and Eastop 1994). *Astegopteryxbambusae* was originally described as *Oregmabambusae* by Buckton (1893) based on samples on *Bambusaarundinacea* in Dehra Dun, India, with the erection of the genus *Oregma*, now a junior synonym of *Astegopteryx* (Buckton 1893; Blackman and Eastop 2018). The original description of the oval-shaped apterous viviparous female was obscure and simple when judged by today’s criteria. Moreover, the description as “color greenish brown, more or less mottled with black” in Buckton (1893) may have been based on dead specimens (Blackman and Eastop 2018). Takahashi (1921) originally described *A.bambucifoliae* (as *Oregmabambucifoliae*) attacking *Bambusa* spp. in Taiwan Island, with yellowish or fresh green body and a distinct character, “a pair of longitudinal dark green patches on the dorsum, which are often interrupted at mid-length” (Takahashi 1921). Later other morphological characters observed in mounted specimens such as the morphology of the wax glands were introduced to distinguish these two species (Noordam 1991; Qiao et al. 2018). For example, in the key to species of *Astegopteryx* of Qiao et al. (2018), wax cells tightly connected or not, and wax cells discernible or not, were used to separate these two species. However, in practice it is still hard to distinguish them due to overlap of morphological characters of different populations. We also observed many times in the field that the occurrence of wax and dark green patches varied across populations in both *A.bambusae* and *A.bambucifoliae*. This indicates that the stability of proposed morphological diagnostic characters for these two species with similar habitats and times of occurrence is uncertain (Blackman and Eastop 2018), leading to doubts about their validity. Further detailed study including wider sampling is necessary to understand more about the morphological variation in both species, and molecular data analysis is crucial to clarify any distinction between them. In addition, considering that the mounting process of aphid slides may discard some useful morphological information, we think that the appearance of live specimens is helpful to understand morphological variation within or between species.

In the present study, based on an extensive sampling effort in subtropical China as well as molecular data from four mitochondrial and nuclear gene markers (cytochrome c oxidase subunit I, COI; cytochrome b, Cytb; tRNA/COII; elongation factor-1α, EF-1α), we aimed to show the spatial and temporal morphological diversity of both species, and test the validity of the two species by integrating the molecular and morphological data.

## Materials and methods

### Sampling

We did extensive field collections in subtropical China (including Fujian, Guangdong, Hainan, Guangxi, Yunnan provinces, ca. 18°15'–27°19'N, 100°15'–120°12'E) from 2015 to 2017. During the field work, photographs of live individuals were taken for all samples using a digital camera (Cannon EOS 7D plus Canon EF 100mm f/2.8L Macro IS USM Lens). Collected specimens were preserved in 95% ethanol and stored at -20 °C for further molecular experiments. The voucher specimens were stored at the Fujian Agriculture and Forestry University. For the final analyses, 37 specimens were chosen to represent the diversity of geography and time as clearly as possible. In accordance with the original descriptions of the two nominal species (Buckton 1893; Takahashi 1921) and other references (Noordam 1991; Blackman and Eastop 2018; Qiao et al. 2018), sixteen samples with an obvious pair of longitudinal dark green patches on the dorsum and relatively narrower body shape were tentatively identified as *A.bambucifoliae*, while 21 samples with relatively broader pear-shaped body and more wax were tentatively determined as *A.bambusae*. Based on current knowledge about the species relationships among this genus and related groups from previous literature (Aoki and Kurosu 1995; Stern et al. 1997; Blackman and Eastop 2018), two specimens of the closely-related but distinct species *A.formosana* were used as outgroups for phylogenetic tree reconstruction. Detailed specimen information including host plant, collection locality, voucher number, and GenBank accession number are shown in Table 1.

**Table 1.** Samples used in this study, with collection information and GenBank accession numbers.

Species (putative designation)	Host plant	Location	Voucher number	Accession number			
				COI	Cytb	EF	tRNA/COII
* Astegopteryx bambucifoliae *	bamboo	Fujian, Fuzhou	HL20160326_4	MH821567			
	bamboo	Fujian, Fuzhou	HL20160326_5	MH821568			
	bamboo	Fujian, Fuzhou	HL20160409_11	MH821537			
	bamboo	Fujian, Fuzhou	HL20160417_7	MH821538			
	bamboo	Fujian, Fuzhou	HL20160512_1	MH821539	MK028307	MK028325	MK372350
	bamboo	Fujian, Fuzhou	HL20161127_3	MH821542			
	bamboo	Fujian, Fuzhou	HL20161127_4	MH821543	MK028308	MK028331	MK372351
	bamboo	Fujian, Fuzhou	HL20161228_18	MH821544			
	bamboo	Guangdong, Shenzhen	HL20170205_7	MH821545	MK028309	MK028332	MK372352
	bamboo	Guangdong, Shenzhen	HL20170205_8	MH821546			
	bamboo	Fujian, Fuding	HL20170403_10	MH821549	MK028310	MK028333	MK372353
	bamboo	Fujian, Fuzhou	HL20170409_2	MH821551			
	bamboo	Fujian, Fuzhou	HL20170409_3	MH821554	MK028311		MK372354
	bamboo	Fujian, Fuzhou	HL20170419_4	MH821556			
	bamboo	Fujian, Fuzhou	HL20170926_23	MH821559	MK028312	MK028334	MK372355
	bamboo	Guangxi, Chongzuo	HLzld20171102_15	MH821571	MK028313		MK372356
* A. bambusae *	bamboo	Fujian, Fuzhou	HL20150416_14	MH821562			
	bamboo	Fujian, Fuzhou	HL20150510_2	MH821570			
	bamboo	Fujian, Fuzhou	HL20150530_4	MH821561			
	bamboo	Fujian, Xiamen	HL20160131_8	MH821563	MK028314	MK028335	MK372357
	bamboo	Hainan, Sanya	HL20160217_1	MH821565	MK028315		MK372358
	bamboo	Fujian, Fuzhou	HL20160308_1	MH821566			
	bamboo	Fujian, Fuzhou	HL20160412_5	MH821569	MK028316	MK028336	MK372359
	bamboo	Fujian, Ningde	HL20161004_1	MH821540	MK028317	MK028337	MK372360
	bamboo	Guangdong, Shenzhen	HL20170205_9	MH821548	MK028318	MK028338	MK372361
	bamboo	Fujian, Fuzhou	HL20170226_3	MH821560			
	bamboo	Fujian, Fuzhou	HL20170318_3	MH821547			
	bamboo	Fujian, Fuding	HL20170403_13	MH821550			
	bamboo	Fujian, Fuzhou	HL20170409_4	MH821555			
	bamboo	Fujian, Fuzhou	HL20170606_8	MH821557			
	bamboo	Yunnan, Kunming	HL20170806_1	MH821558	MK028319		MK372362
	bamboo	Guangxi, Chongzuo	HLzld20171103_22	MH821572			
	bamboo	Yunnan, Kunming	HLzld20171108_6	MH821573	MK028320	MK028326	MK372363
	bamboo	Yunnan, Kunming	HLzld20171108_7	MH821574			
	bamboo	Yunnan, Kunming	HLzld20171111_3	MH821576	MK028321	MK028327	MK372364
	bamboo	Yunnan, Dali	HLzld20171126_6	MH821577			
	bamboo	Yunnan, Dali	HLzld20171126_7	MH821578	MK028322	MK028328	
* A. formosana *	bamboo	Guangxi, Chongzuo	HLzld20171102_16	MH821579	MK028323	MK028329	
	bamboo	Guangxi, Chongzuo	HLzld20171103_19	MH821582	MK028324	MK028330	MK372365
A.bambucifoliae*		Guizhou	ZMIOZ13322	JN032708		DQ493848	
A.bambusae*	* Bambusa tulda *	India, Karnataka	ORP-2010-61	HQ112196			
		Guangxi	ZMIOZ 14592	JX282768	JX282692	JX282849	
	* Bambusa tulda *	India, Bangalore	KBRIIHR-172	JX051408			
	* Bambusa tulda *	India, Karnataka	KBRIIHR-149	JX051385			
	* Bambusa tulda *	India, Karnataka	KBRIIHR-148	JX051384			
	* Bambusa tulda *	India, Karnataka	KBRIIHR-147	JX051383			
	* Bambusa tulda *	India, Karnataka	KBRIIHR-146	JX051382			
* A. bambucifoliae *	Poaceae	Taiwan, Puli					L27324
A.formosana*	Poaceae	Taiwan, Sun Moon Lake					L27326

* indicates the sequences downloaded from the GenBank.

### DNA extraction, PCR, and sequencing

We used DNeasy Blood &Tissue Kit (QIAGEN, GERMANY) to extract total genomic DNA from one individual per sample. The primers LepF (5’-ATTCAACCAATCATAAAGATATTGG-3’) and LepR (5’-TAAACTTCTGGATGTCCAAAAAATCA-3’) (Foottit et al. 2008) were used to amplify COI barcode region. The primers for amplification of Cytb were CP1 (5’-GATGATGAAATTTTGGATC-3’) and CP2 (5’-CTAATGCAATAACTCCTCC-3’) (Harry et al. 1998). EF-1α sequences were amplified based on EF3 (5’-GAACGTGAACGTGGTATCAC-3’) and EF2 (5’-ATGTGAGCAGTGTGGCAATCCAA-3’) (Palumbi 1996; von Dohlen et al. 2002). tRNA/COII sequences were amplified based on mt2793 + (5’-ATACCTCGACGTTATTCAGA) and mt3660- (5’- CCACAAATTTCTGAACATTGACCA) (Stern 1994). The PCR was performed in 30 μl reaction volumes: 20 μl ddH2O, 3 μl 10Xbuffer, 2.4 μl dNTP, 0.6 μl forward and reverse primer (10 μM), 0.4 μl of Taq DNA polymerase (5U/μl) and 3 μl of template DNA. All polymerase chain reactions included an initial denaturation step for 5 min at 95 °C and final extension step for 10 min at 72 °C. The cycling conditions of COI included 35 cycles of denaturation at 94 °C for 20s, annealing at 50 °C for 30s and extension at 72 °C for 2 min. The cycling conditions for Cytb were: 35 cycles of 1 min at 92 °C, 1.5 min at 48 °C and 1 min at 72 °C. The thermal setup for EF-1α was: 35 cycles of 30s at 95 °C, 1 min at 51 °C and 1 min at 72 °C. The cycling conditions for tRNA/COII were 34 cycles of 30s at 95 °C, 1 min at 54 °C and 1 min at 72 °C. Detection of the PCR products was performed on a 1% agarose gel. The eligible products were bidirectionally sequenced using the same PCR primer pairs by Sangon Biotech (Shanghai).

### Sequence and phylogenetic analyses

Thirty-nine COI sequences were successfully obtained from the 37 ingroup samples and two *A.formosana* outgroups. In addition, eight COI sequences including one of *A.bambucifoliae* and seven of *A.bambusae* were downloaded from GenBank (accession numbers: JN032708, HQ112196, JX282768, JX051408, JX051385, JX051384, JX051383 and JX051382) for further phylogenetic analyses (Table 1). Based on the topology of the COI tree, sixteen ingroup samples were selected for Cytb, tRNA/COII, and EF-1α amplification. Finally, a total of 16 Cytb sequences, 12 EF-1α sequences and 15 tRNA/COII sequences were successfully generated. We downloaded several Cytb (accession number: JX282692) and EF-1α (accession numbers: DQ493848, JX282849) sequences of both species from the GenBank. Furthermore, as *A.bambucifoliae* was originally described from Taiwan, we downloaded two tRNA/COII sequences L27324 (*A.bambucifoliae*) and L27326 (*A.formosana*), which were obtained from Taiwanese samples from GenBank to test the relationships between them and our sequences (Table 1). For all the sequences obtained in this study, the raw forward and reverse sequences were corrected based on the chromatograms and assembled using BioEdit software (Hall 1999). Subsequently, the sequences were aligned by MAFFT (Kazutaka and Standley 2013) and trimmed to the same length with BioEdit. For the EF-1α sequences, the introns were removed according to the GT-AG rule and the cDNA region of a *Schizaphisgraminum* reference sequence (GenBank accession number AF068479), and the coding regions of EF-1α were used in further phylogenetic analyses.

The Kimura 2-parameter (K2P) model (Kimura 1980) were used to calculate pairwise distances among nucleotide sequences in MEGA 7.0 (Kumar et al. 2016). The optimal nucleotide substitution models were determined based on Akaike Information Criterion (AIC) by using jMODELTEST 2.1.7 (Darriba et al. 2012) for COI (GTR+I), Cytb (GTR), EF-1α (HKY+I) and tRNA/COII (GTR). For each marker, different phylogenetic reconstruction methods (Neighbor-joining, NJ; Maximum likelihood, ML; Bayesian inference, BI) were used to estimate the topologies. MEGA 7.0 was used to build the NJ trees based on the K2P model and 1,000 bootstrap replicates. Based on the estimated models, the ML trees were estimated in RAxML (Silvestro and Michalak 2012) with the settings of ML+ rapid bootstrap, and nodal support was calculated by 1000 replicates. The Bayesian analyses were performed with MrBayes 3.2.6 (Ronquist et al. 2012). Two million generations Markov Chain Monte Carlo (MCMC) were run and sampled every 100 generations, and the first 25% of trees were discarded as burn-in to acquire posterior probability values (PP). The phylogenetic trees were represented and edited using the online tool iTOL (Letunic and Bork 2016).

The haplotype network analysis of COI sequences was also implemented to illustrate the population genetic structure in space based on geographic groups. The COI sequences were imported into DNAsp 5.0 (Librado and Rozas 2009) to analyze the haplotype composition. Then the median-joining network of the haplotypes was computed by using NETWORK 5.0.0.3 (Bandelt et al. 1999) based on default settings.

## Results

### Sequence characters

Forty-seven COI sequences were aligned to a final length of 556 bp, which included 527 conserved sites, 29 variable sites, and 24 parsimony-informative sites. The nucleotide composition of COI alignment displayed a strong bias toward A+T content (T: 42.6%, C: 12.7%, A: 36.2% and G: 8.5%). The 718 bp long Cytb alignment with 19 sequences included 689 conserved sites, 29 variable sites, and 28 parsimony-informative sites. The nucleotide composition of Cytb alignment was 44.8% T, 12.3% C, 34.2% A, and 8.7% G. After the introns were excluded, sixteen EF-1α sequences were trimmed to a 785 bp long alignment with 769 conserved sites, 16 variable sites, and 13 parsimony-informative sites. The nucleotide composition was 26.2% T, 20.9% C, 27.8% A, and 25.1% G. The tRNA/COII alignment had 626bp with 595 conserved sites, 31 variable sites and 25 parsimony-informative sites. The nucleotide composition of tRNA/COII alignment was 41.0% T, 11.1% C, 41.1% A, and 6.8% G.

### Genetic distances and phylogenetic analyses

The intraspecific and interspecific K2P genetic distances among the samples are shown in Table 2. The maximum genetic distances (1.46%) were between some Indian samples and the other samples. Basically, the COI sequences were able to contribute more informative sites to understand the population structure.

**Figure 1.**
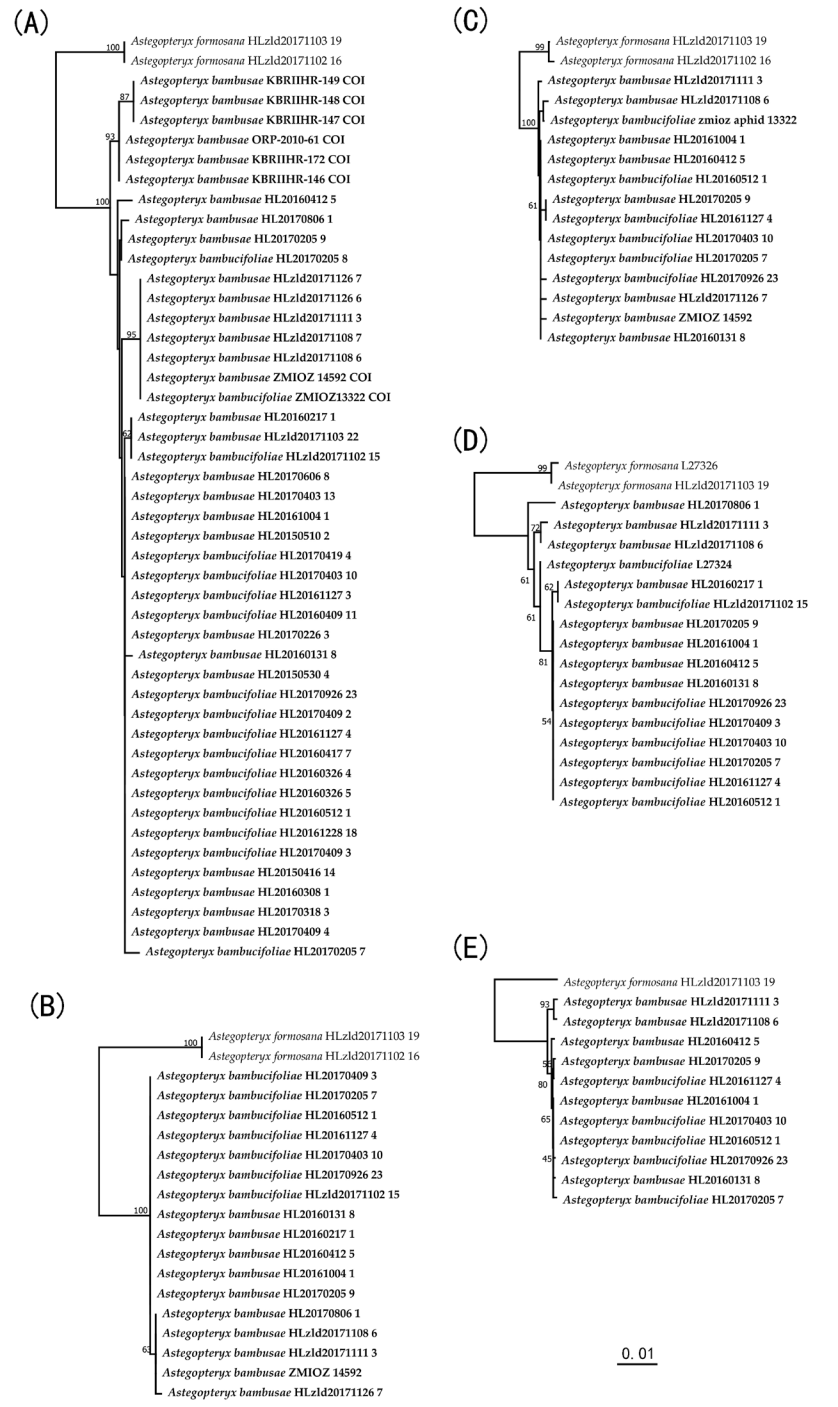
The Neighbor-joining (NJ) trees based on COI (A), Cytb (B), EF-1α (C), tRNA/COII (D), and the combined data of all four genes (E). The ingroup specimens are printed in bold and the bootstrap values higher than 50 are indicated. The sequences are named as putative species name plus specimen voucher number.

In general, different reconstruction approaches yielded similar phylogenetic trees for the same marker (Figure 1, Suppl. materials 1, 2). Phylogenetic trees showed that all four genes failed to support the monophyly of both *A.bambucifoliae* and *A.bambusae*. Samples of these two species were dispersed in different clades of the phylogenetic trees. Based on the COI tree with more samples (Figure 1), some well-supported clades were distinct. All the samples from the Yunnan and Guizhou plateau of southwestern China as well as all the Indian samples clustered into separate clades. These samples were all morphologically identified as *A.bambusae*. There was also a separate clade including many samples of both morphologically identified species and from different localities of southeastern and southern China, but with low genetic distances.

**Figure 2.**
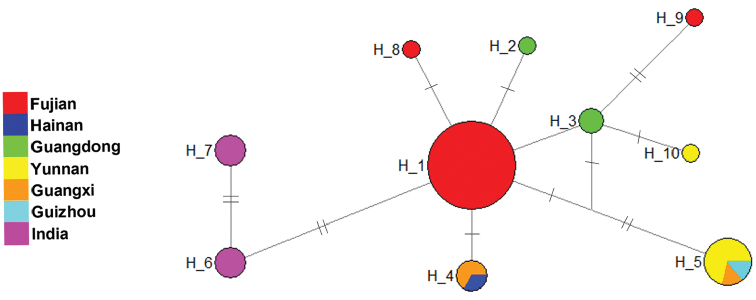
Haplotype networks based on COI sequences. The circles represent different haplotypes, while different colors correspond to the geographical origins of samples and sizes represent relative numbers of sequences (H_1: 23; H_2: 1; H_3: 2; H_4: 3; H_5: 7; H_6: 3; H_7: 3; H_8: 1; H_9: 1; H_10: 1). The short line segments indicate mutated positions between haplotypes.

The network analysis of the COI haplotypes (Figure 2) indicated that all the Indian samples, assigned as haplotypes H6 and H7, were linked together and showed greatest differentiation from the other haplotypes.The samples from southwestern China, including almost all samples from Yunnan and Guizhou Plateau and some from Guangxi, were of haplotype H5. Haplotype H1 with most samples included almost all samples from Fujian in southeastern China. The other samples from southeastern and southern China (Fujian, Guangdong, Hainan) were assigned to several other haplotypes, i.e., H8, H9, H2, H3, H4.

**Table 2.** Genetic distances among *Astegopteryxbambucifoliae* and *A.bambusae* samples based on COI, Cytb, EF-1α, and tRNA/COII sequences.

Genetic distance	Species	Gene	Range (%)	Mean (%)
Intraspecific	* Astegopteryx bambucifoliae *	COI	0–0.91	0.15
		Cytb	0	0
		EF-1α	0–0.26	0.13
		tRNA/COII	0–0.48	0.12
	* Astegopteryx bambusae *	COI	0–1.46	0.56
		Cytb	0–0.28	0.11
		EF-1α	0–0.38	0.19
		tRNA/COII	0–1.46	0.61
Interspecific	*Astegopteryxbambucifoliae* & *Astegopteryxbambusae*	COI	0–1.46	0.38
		Cytb	0–0.28	0.08
		EF-1α	0–0.38	0.14
		tRNA/COII	0–1.46	0.38

### Phylogenetic pattern of morphological variation

**Figure 3.**
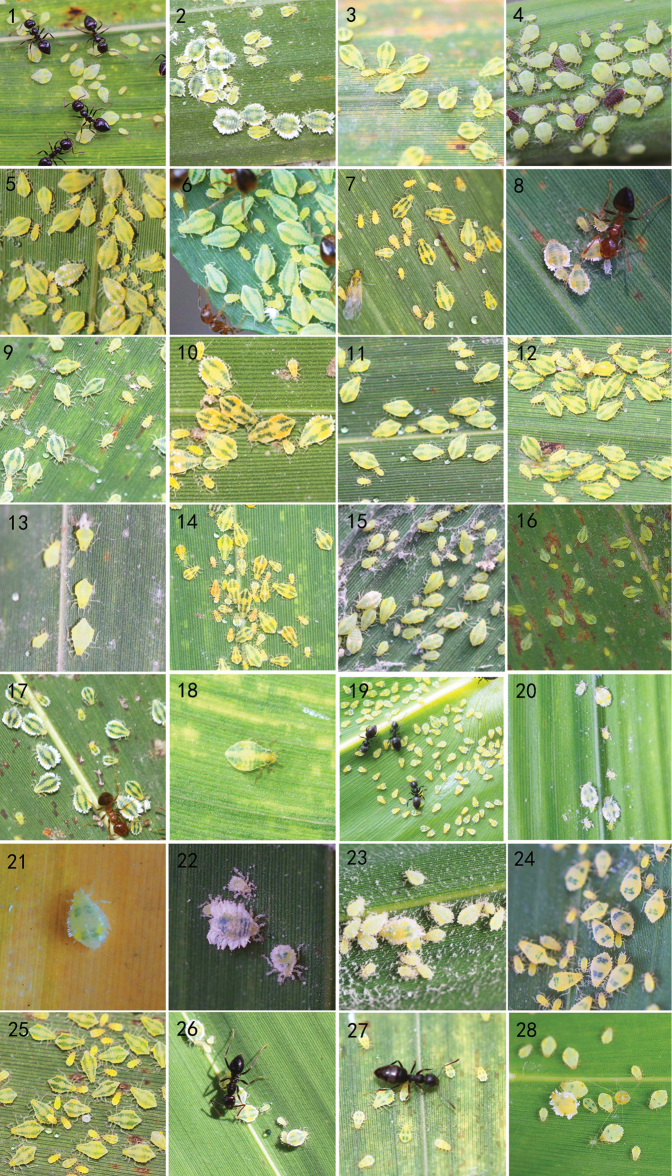
Photographs of live specimens showing high morphological variation among samples. Based on specimen voucher number, these photographs correspond to the following sequences in the phylogenetic trees; **1** HL20170205_7 **2** HL20170606_8 **3** HL20170409_2 **4** HL20170403_13 **5** HL20170226_3 **6** HL20150416_14 **7** HL20160417_7 **8** HL20161004_1 **9** HL20161228_18 **10** HL20150530_4 **11** HL20160326_4 **12** HL20170403_10 **13** HL20160131_8 **14** HL20160512_1 **15** HL20170318_3 **16** HL20170419_4 **17** HL20170926_23 **18** HL20160217_1 **19** HLzld20171102_15 **20** HLzld20171103_22 **21** HLzld20171108_6 **22** HLzld20171108_7 **23** HLzld20171111_3 **24** HLzld20171126_6 **25** HL20170205_8 **26** HL20170806_1 **27** HL20160412_5 **28** HLzld20171102_16.

The photographs of live specimens that we took during the field work in different localities and at different times indicated the spatial and temporal diversity of all samples (Figure 3). When these photographs were compared with the phylogenetic tree (Figure 1), it was apparent that some key morphological diagnostic characters used to distinguish both species, such as the wax types and the green patches, have no distinct phylogenetic pattern. For example, within the separate clade including many samples of both morphologically identified species from different localities of southeastern China (Figure 3[1–17]), the appearance of these samples based on wax layout and green patches varied greatly, whereas their genetic distances were very low. Moreover, although the samples from Yunnan Plateau with identical COI sequence (Figure 3[21–24]) had relatively similar green patches and were collected at similar times (November 2017), their wax density and distribution were clearly different.

## Discussion

Species descriptions based on limited samples are often unable to represent the whole picture of morphological variation within the species, making it likely that some names will subsequently be synonymised (Winston 1999; Eastop and Blackman 2005). A review of the relevant literature and the results of our present study indicate that *A.bambusae* and *A.bambucifoliae* should be such a case. Based on the molecular data from extensive sampling, our results show that relatively low genetic distances of four genes exist among all samples of both morphologically identified species. In previous DNA barcoding studies of aphids (Foottit et al. 2009; Zhu et al. 2017), 2% has been used as a threshold value of COI genetic distances for species delimitation. This threshold has also been proposed for other insect groups (Hajibabaei et al. 2006; Zahiri et al. 2014). In the present study, the maximum and mean COI genetic distances (1.46% and 0.56%, respectively; Table 2) among all samples from southern China to India do not reach the 2% threshold value to define separate species. Moreover, no matter what phylogenetic methods were used, the monophyly of neither of the morphologically identified *Astegopteryx* species has been supported by the phylogenetic trees based on any of the four genes. Although all ingroup samples form one well-supported clade, several inner clades with dispersed samples of both species have been less supported with lower bootstrap values. Thus, the molecular data indicate that all samples belong to a single species.

Our study also provides information on the taxonomic significance of variations in appearance in life. Results show that there is no distinct phylogenetic pattern for key diagnostic characters such as green patches on the dorsum and distribution of wax. The high spatial and temporal morphological diversity among all samples used in the present study support our and other colleagues’ speculation (Blackman and Eastop 2018) that the stability of these proposed morphological discriminants for the two *Astegopteryx* species is uncertain. The distinct character of a pair of longitudinal dark green patches often interrupted at mid-length on the dorsum of live specimens was proposed by Takahashi (1921) to distinguish *A.bambucifoliae*. However, this character has been described as “uninterrupted longitudinal markings on dorsum” by other taxonomists (Joshi and Poorani 2007), indicating that this character cannot be a stable diagnostic character at species level. Wax gland plates occur widely in the subfamily Hormaphidinae, which *Astegopteryx* belongs to, and have a variety of shapes and sizes as well as complex arrangements (Chen and Qiao 2012). Previous studies showed that characters related to wax gland plates even change ontogenetically, for example, wax gland plates may be present in nymphs and embryos but absent in adults (Shaposhnikov and Gabrid 1987). Considering aphids are producing honeydew and Cerataphidini aphids often live as large colonies in wet subtropical regions (Noordam 1991; Huang et al. 2012; Blackman and Eastop 2018; Qiao et al. 2018), the wax probably has a functional role to protect aphids from possible contamination of honeydew, rain, natural enemies, and other environmental factors (Pope 1983; Heie 1987; Smith 1999; Pike et al. 2002; Moss et al. 2006). Such a functional character may not necessarily be phylogenetically informative for species delimitation, as the appearance and arrangement of wax cells may be easily affected by environmental changes. This is shown by the high wax variation among all samples showed in the present study.

By integrating the molecular data and morphological information, our results indicate that *A.bambusae* and *A.bambucifoliae* should be regarded as a single species with high intraspecific morphological variation. Based on the history of the two species, we place *A.bambucifoliae* (Takahashi 1921) as a junior synonym of *A.bambusae* (Buckton 1893). Considering the results of our study, as well as published descriptions (Buckton 1893; Takahashi 1921; Noordam 1991; Qiao et al. 2018), it seems that large-scale geographic patterns of population differentiation may exist within the species. For example, the Indian samples we cited seem more genetically divergent. Noordam (1991) reviewed the Javanese *Astegopteryx* species, in which several species originally described by van der Goot (1917) were considered as color varieties of *A.bambusae*. However, based on the color plates (Pl. 1–5) of live specimens in Noordam (1991), the patterns of green bands and wax distribution of those color varieties are quite different from our photographed specimens. This may raise the question of whether the treatment in Noordam (1991) is appropriate. Therefore, future investigation is needed to resolve the identity of populations in Southeast Asia. In addition, considering this species has previously been recorded with facultative host alternation between primary host *Styrax* and secondary host bamboos in Taiwan (Aoki and Kurosu 2010), it will be interesting to have some molecular work done in future on populations from *Styrax*.

## Supplementary materials

Supplementary material 1 The Maximum likelihood (ML) trees based on COI (A), Cytb (B), EF-1α (C), tRNA/COII (D) and the combined data of all four genes (E) Data type: molecular data Explanation note: The ingroup specimens are printed in bold and the bootstrap values over 50 are indicated. The sequences are named as species name plus specimen voucher number. This dataset is made available under the Open Database License (http://opendatacommons.org/licenses/odbl/1.0/). The Open Database License (ODbL) is a license agreement intended to allow users to freely share, modify, and use this Dataset while maintaining this same freedom for others, provided that the original source and author(s) are credited.Qiang Li, Jiamin Yao, Lingda Zeng, Xiaolan Lin, Xiaolei Huang

Supplementary material 2 The Bayesian trees based on COI (A), Cytb (B), EF-1α (C), tRNA/COII (D) and the combined data of all four genes (E) Data type: molecular data Explanation note: The ingroup specimens are printed in bold and the posterior probabilities over 90% are indicated. The sequences are named as species name plus specimen voucher number. This dataset is made available under the Open Database License (http://opendatacommons.org/licenses/odbl/1.0/). The Open Database License (ODbL) is a license agreement intended to allow users to freely share, modify, and use this Dataset while maintaining this same freedom for others, provided that the original source and author(s) are credited.Qiang Li, Jiamin Yao, Lingda Zeng, Xiaolan Lin, Xiaolei Huang
