# Supplementary figures and images for: Molecular and morphological evidence for the identity of two nominal species of Astegopteryx (Hemiptera, Aphididae, Hormaphidinae)
Source: Zookeys. 2019 Mar 25;833:59–74. doi: 10.3897/zookeys.833.30592 (PMC6443623; doi:10.3897/zookeys.833.30592)

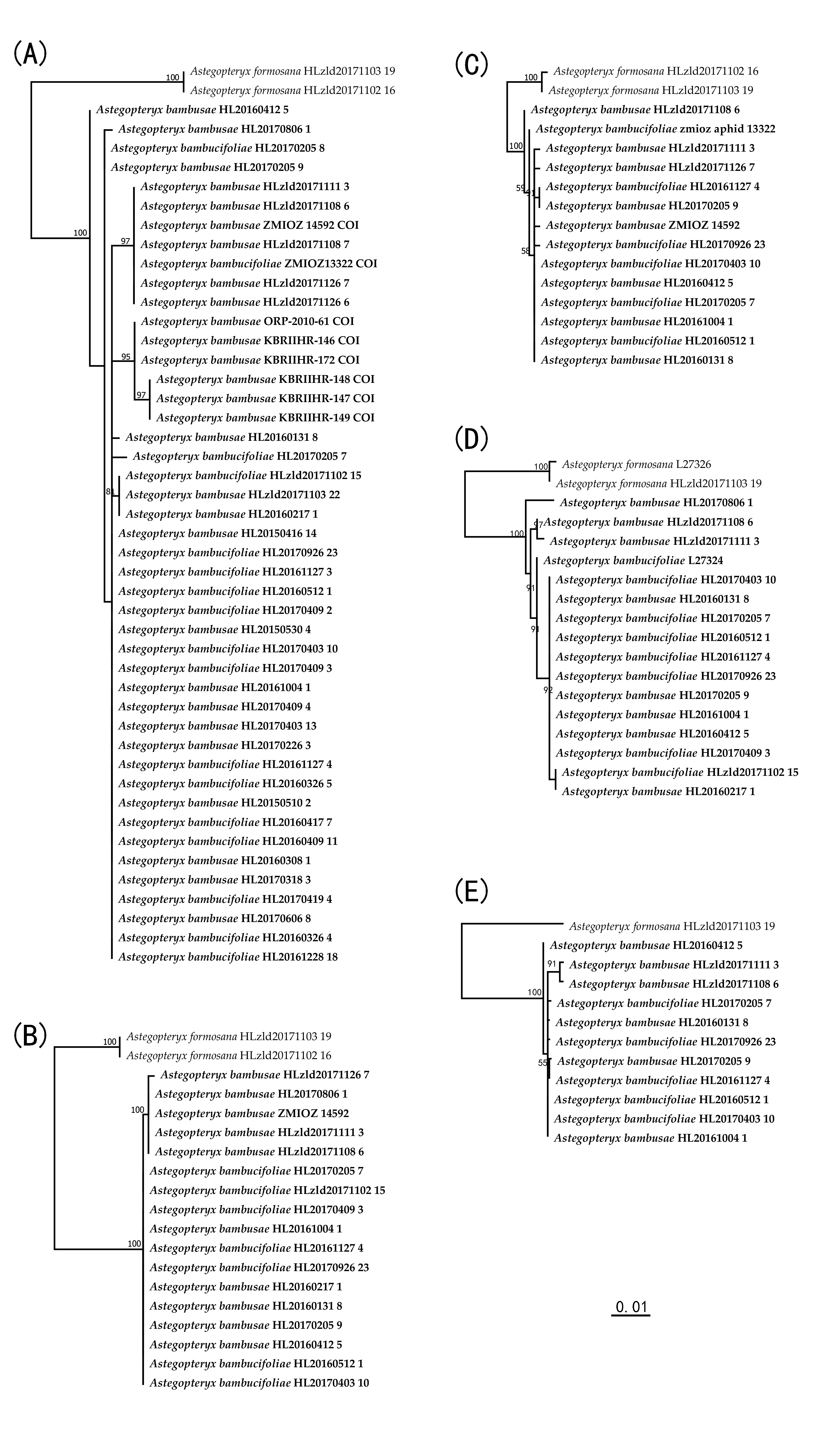

Supplement: Supplementary material 1 [file zookeys-833-059-s001.jpg]

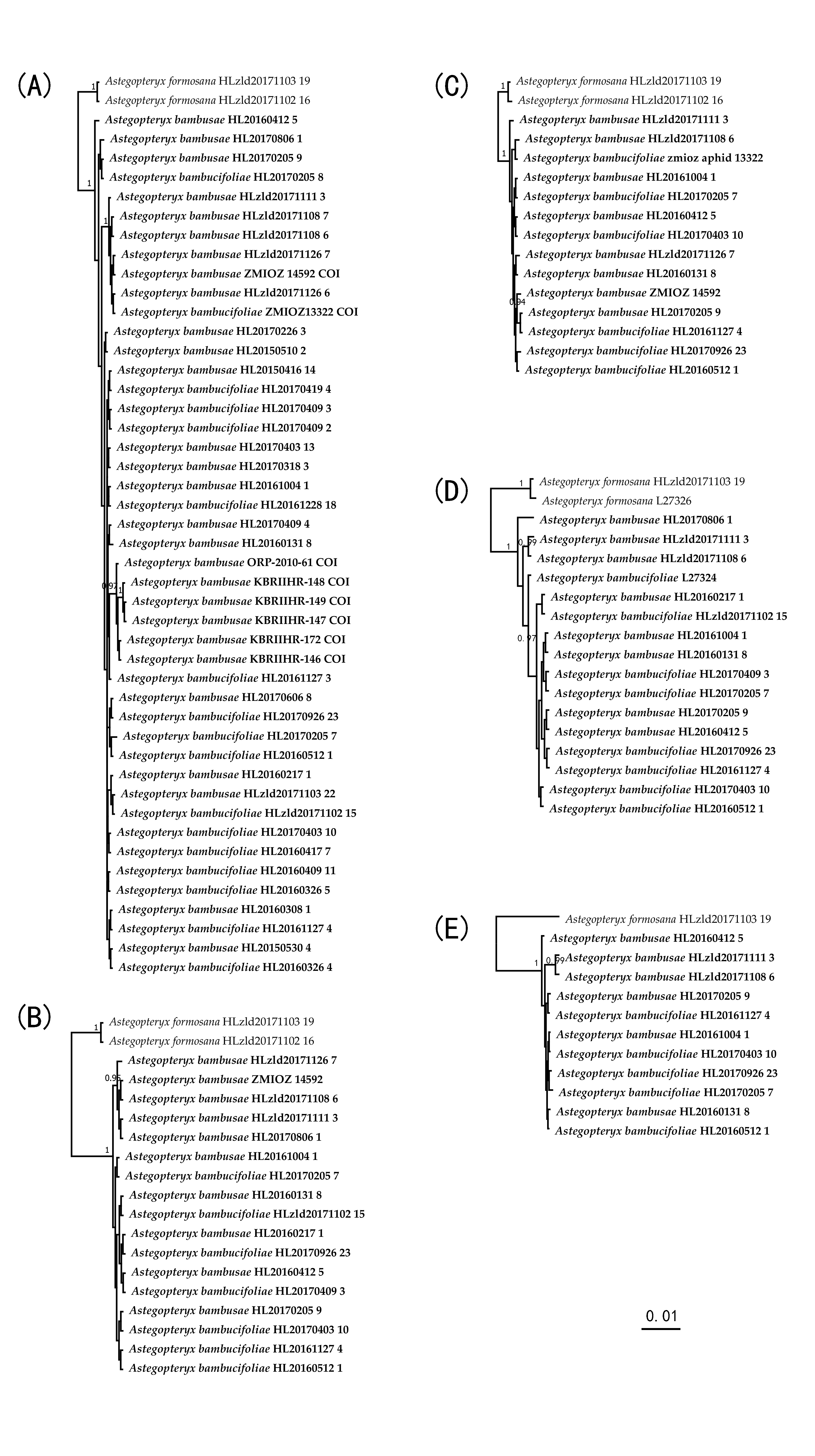

Supplement: Supplementary material 2 [file zookeys-833-059-s002.jpg]
